# Is Empiricism Empirically False? Lessons from Early Nervous Systems

**DOI:** 10.1007/s12304-017-9294-7

**Published:** 2017-06-27

**Authors:** Marcin Miłkowski

**Affiliations:** Institute of Philosophy and Sociology, Polish Academy of Sciences, ul. Nowy Świat 72, 00-330 Warszawa, Poland

**Keywords:** Empiricism, Skin-brain thesis, Cognitive activity, Sensorimotor organization

## Abstract

Recent work on skin-brain thesis (de Wiljes et al. 2015; Keijzer 2015; Keijzer et al. 2013) suggests the possibility of empirical evidence that empiricism is false. It implies that early animals need no traditional sensory receptors to be engaged in cognitive activity. The neural structure required to coordinate extensive sheets of contractile tissue for motility provides the starting point for a new multicellular organized form of sensing. Moving a body by muscle contraction provides the basis for a multicellular organization that is sensitive to external surface structure at the scale of the animal body. In other words, the nervous system first evolved for action, not for receiving sensory input. Thus, sensory input is not required for minimal cognition; only action is. The whole body of an organism, in particular its highly specific animal sensorimotor organization, reflects the bodily and environmental spatiotemporal structure. The skin-brain thesis suggests that, in contrast to empiricist claims that cognition is constituted by sensory systems, cognition may be also constituted by action-oriented feedback mechanisms. Instead of positing the reflex arc as the elementary building block of nervous systems, it proposes that endogenous motor activity is crucial for cognitive processes. In the paper, I discuss the issue whether the skin-brain thesis and its supporting evidence can be really used to overthrow the main tenet of empiricism empirically, by pointing out to cognizing agents that fail to have any sensory apparatus.

## Introduction: The Debate over the Function of the Nervous System

In this paper, I stress the importance of the debate over the evolution of the nervous system for epistemological questions. While the received view has it that the nervous system first evolved to make organisms respond to environmental stimuli via more and more complex, integrated systems of stimulus-response pathways, there is evidence that the evolutionary origin of the nervous system had more to do with the control function of the nervous system. This may comprise motor control or metabolic control. Interestingly, the alternative account of the initial adaptive function of the nervous system is more difficult to reconcile with a traditional claim of empiricism that all knowledge starts from sensation (I will analyze the core claim in more detail in Empiricism and Minimal Cognition section). Note that the alternative account does not claim that the original function of the nervous system has remained the same; its proponents assume that the proper function of the nervous system has changed over time and in most organisms, it includes responding to sensory stimuli.

The purpose of my argument is both to highlight that, in principle, the traditional philosophical claim of empiricism might be empirically disconfirmed, and to show that current evolutionary biology and neuroscience, along with biosemiotics, have an important role in highlighting and analyzing functions of mechanisms underlying cognition. It’s quite implausible to suppose that a single paper might overthrow a huge philosophical tradition, and indeed, I think there are many ways empiricists may respond and qualify their claims. However, there is a conceptual space to explore.

In the argument, I will assume a broadly naturalistic approach to cognition or knowledge, as is usual in biosemiotics. For this reason, I will assume that there is a kind of cognition that does not require human agents, human language, entertaining propositions consciously, and such. Hence, non-naturalist or anthropocentric approaches to knowledge or cognition are not targeted by my argument at all.

## Empiricism and Minimal Cognition

Even though empiricism is one of the most important views in contemporary philosophy, it’s notoriously difficult to find attempts to succinctly define its core claim (Humphreys 2004, p. 10). I will focus only on the sensory-related claims of empiricists; in other words, my argument pertains *only* to empiricism as related to sense experience. There are more sophisticated versions of empiricism that arguably go beyond sensory receptors, like ‘scientific empiricism’ defended by Paul Humphreys, who stresses that human senses are not the basis for scientific endeavors, as the senses miserably fail to function reliably, in contrast to our measuring instruments. Paul Feyerabend argued similarly (Feyerabend 1969). The argument defended here is not supposed to argue against such brands of empiricism. At the same time, the argument is directed not only against empiricism but also against some of its critics from the Kantian tradition, pragmatism, or ecological psychology, who all accept the primacy of sensory processing for cognition. In other words, I focus on the purported epistemic primacy of sensation.

The claim of empiricism may be formulated as related to knowledge or meaning. In epistemology, the claim is:

(EE) All knowledge is ultimately based on sense experience.

In theory of meaning, it boils down to:

(EM) The meaning of all words or concepts is derivative from sense experience.

An important caveat: the empiricist may have no interest in the notion of conscious experience, so, at least in principle, he or she may replace the notion of experience with the notion of data or information, or the given.

The claim (EE) or (EM) is usually based on a certain account of epistemic hierarchy or stratification (hence the predicate ‘ultimately based on’ or ‘derivative from’ in these claims). It is supposed to apply straightforwardly to the basic stratum of knowledge or meaning. Simply, it seems quite intuitive that the basic stratum of knowledge is delivered by perception, and perception relies on the senses. How could it be otherwise?

The two main counterarguments, historically, against empiricism, are the following. First, it may be claimed that there is a priori knowledge not derivable from sense experience. Even if empiricists all agree that mathematical propositions are true, they traditionally argued that such propositions were analytical, or true in virtue of the meaning of terms involved rather than of how the world is. However, Immanuel Kant has argued that there is non-analytic knowledge, which is still a priori (independent from sense experience with respect to justification). This started probably the most important strand in the criticism of empiricism, even if in the contemporary discussions, under the influence of arguments presented by Quine (1976), the importance of the analytic/synthetic distinction has faded. However, there are still followers of the Kantian tradition who stress that there is some special, non-empirical knowledge about the realm of reasons, pure normativity and such. In spite of this, the status of such knowledge remains controversial, and empiricists may remain unmoved by such arguments. Still, for naturalistic approaches to cognition, there are plausible ways to explicate a priori knowledge in biological terms, as proposed by Konrad Lorenz (1941) and other proponents of evolutionary epistemology (Campbell 1974). However, the debate on the innate knowledge or concepts is quite complex (Samuels 2002), and even if there is a way to elucidate this notion in a biologically and naturalistically plausible way, it will not be of my concern here.

The second counterargument is an attempt at self-refutation. It claims that (EE), and in particular (EM), are not derived from sense experience themselves, as already Kant and many others noted (e.g., Ingarden 1936). A simple defense of empiricism would be to say that this claim does not express any knowledge but a terminological decision, which is itself not totally arbitrary but justified by the non-existence of real counterexamples to the claim. In other words, this objection can still be avoided. What is important for my purposes is that it makes the status of (EE) or (EM) explicit. It may be either (1) a lexical definition that states a necessary truth whose negation should engender a contradiction, as with all conceptual truths (as assumed by Ingarden in his criticism), or (2) a stipulative definition, which cannot be purely arbitrary on pain of being dogmatic. In other words, there must be some reason to accept this definition.

In contrast to these traditional arguments, I demonstrate below how a posteriori (synthetic) knowledge may be accessible, in principle, to some cognitive agents, and remain independent from *sense* experience. In some ways, my argument is similar to Feyerabend’s argument that sensory experience is not necessary for science (a more explicit version of this argument was offered later by Fodor (1991)). What I claim is that sensory experience, or even any reliance on the senses, is not required to make a piece of information knowledge for a cognitive agent. The argument appeals to the skin-brain thesis defended by Fred Keijzer (Keijzer 2015; Keijzer et al. 2013); the thesis claims that the nervous system evolved for motor control, and that organisms with early nervous systems cognize without any sense receptors. They just have motor effectors. Hence, the claim (EE) as a conceptual claim turns out to be false, and as a terminological decision, it is unmotivated. It’s simply conceivable that there are organisms without sense experience but with knowledge. In other words, cognition (which I understand as producing knowledge) is possible without sense experience.

To make my argument, I will assume that evolutionarily early organisms were capable of minimally cognitive processing. This may sound abhorrent to some, but there is already some research on cognitive features of bacteria (van Duijn 2006) and plants (Garzón 2007). Indeed, the same assumption abounds in biosemiotics in general, e.g., Gennaro Auletta (2011) has insisted that one can talk of cognitive biology that starts from bacteria. Some, however, stressed that there might be no overarching understanding of the notion of *cognition*, and maybe there’s no need for a unified understanding (Miłkowski 2013). The situation is similar to that in the life sciences: there is no single overarching notion of life in these sciences, but it does not really affect them negatively at all (Machery 2011). Different disciplines may need to look at cognition, or life, from different perspectives, for different explanatory purposes, and using different idealizations or experimental protocols. As long as there is no danger of duplicating the research effort and confusing cross-talk between disciplines, and ways to identify the crucial phenomena in these sciences, there’s no deep problem here. Instead of giving an account of *all* cognition, I want to focus on some *minimally* cognitive phenomena that may fall short of supporting all possible cognitive processing. The focus on minimal cognition stems from the tradition of evolutionary epistemology, in which the current paper may be also situated. Evolutionary epistemology studies the origins and development of cognitive phenomena in light of biological and cultural evolution.

However, there is a substantial debate among different theoretical frameworks of minimal cognition. For example, Calvo Garzón (2007) proposes to understand cognitive systems as representational information-processing systems. This is a clear proposal, mostly because the notion of information-processing has been made recently quite sophisticated and precise (Fresco 2014; Miłkowski 2013; Piccinini 2015). But many theorists have argued that animals capable only of tropisms or taxes have no representational states (Burge 2010; Miłkowski 2015). Moreover, under Calvo’s proposal, a representational system in control of nothing over and above mere computation counts as cognitive. Arguably, only systems that are in control of behavior, physiology, or development in animals may be explanatory of their behavior, physiology, or development; otherwise, it makes little sense to appeal to computational systems to explain these features of animals. Hence, Calvo’s proposal would imply the existence of an awkwardly epiphenomenal kind of cognition.

Others have stressed that cognition is sensorimotor coordination (van Duijn 2006). I deny that you need sensory information to be cognitive, so I cannot adopt this definition, and I believe that it presupposes a little too much. Instead, I adopt a notion defended by Barandiaran and Moreno (2006): ‘it is not until the adaptive preservation of the internal organization of neural dynamics becomes the major source of neurodynamic regulation that cognition appears’ (Barandiaran and Moreno 2006, p. 180). In other words, as long as there is some adaptive preservation of the internal organization, realized by something functionally equivalent to neural dynamics, we have minimal cognition. This notion is somewhat close to the concept of *minimal mind* as defended by Sharov (2013) but it is not required that biological agents be able to classify objects; in Sharov’s terminology, some minimally cognitive agents could remain protosemiotic, they may include only information in direct control of their actions.

Let me make it clear: this characterization of cognition may be insufficient in other contexts, and it has been claimed that, in comparative psychology, the notion of *cognition* forms an interesting stable cluster of interdependent properties (Buckner 2015). The evolutionarily early systems that I describe clearly fail to be cognitive in the sense that Buckner gives; though they may be context-sensitive, or behave appropriately in different situations, and with appropriate speed, they may fail to form any interesting categories, and surely cannot learn abstract concepts, use multimodally integrated information etc.

## Skin-Brain Thesis

**Fig. 1:**
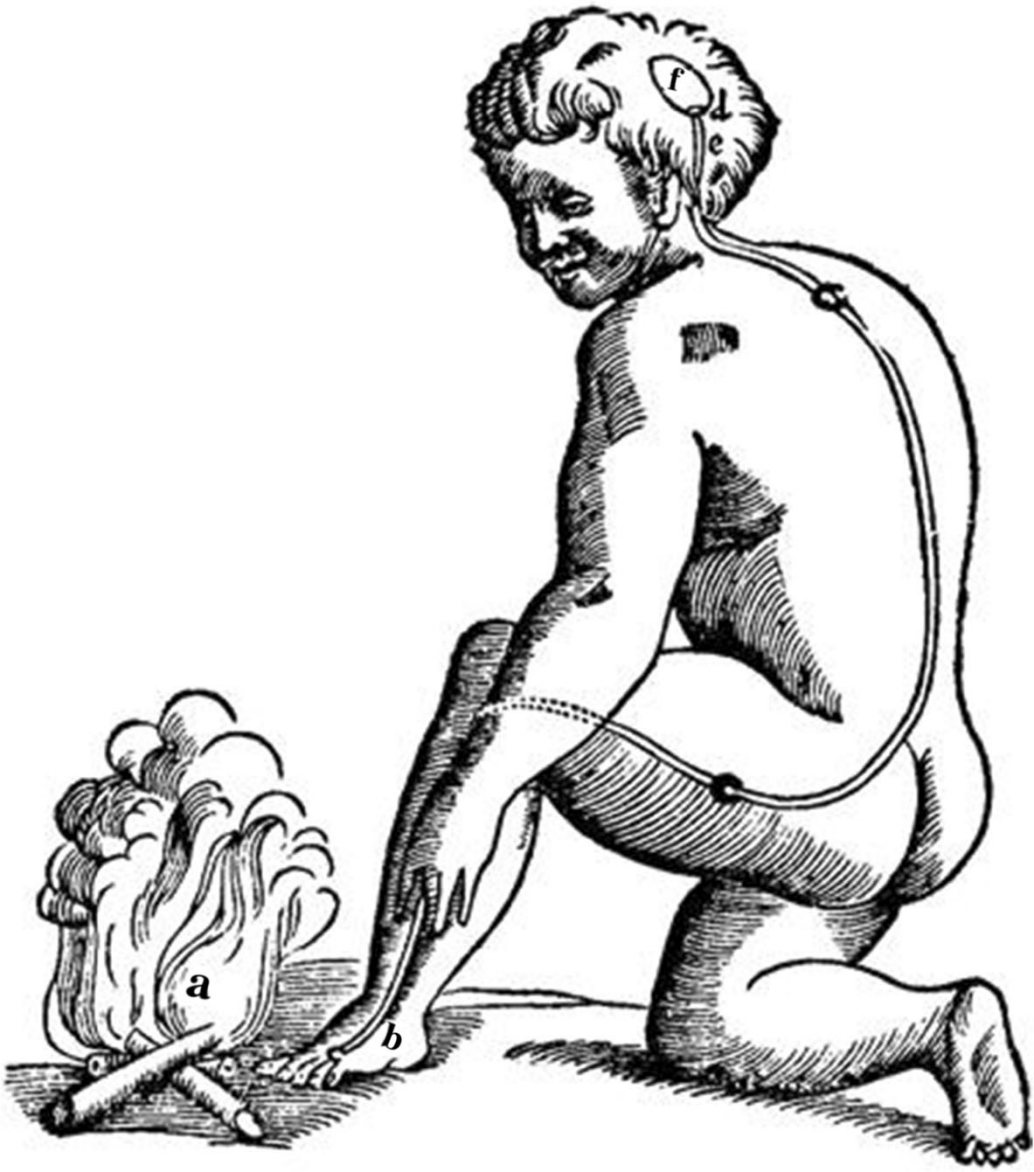
The reflex arc of pain according to Descartes. The fire (*a*) is a stimulus afflicting the skin (*b*) and moving the fine thread (*c*), which goes to valves (*d*, *e*). The valves open the cavity (*f*), from which an animal spirit is released, which in turn makes the head turn and move the hand and the foot

The received view in the evolution of the nervous system is that the nervous system evolved from its basic building block: a reflex arc. This notion can be traced back to Descartes, who offered early mechanistic accounts of the nervous system and psychological phenomena. Descartes conceived of the nervous system in terms of flows of animal spirits. These are produced in the blood and may exert influence over bodily parts. The basic building block of the nervous system, according to Descartes, is the *reflex arc*: the stimulus pulls tiny wires of the nervous system, which in turn open little valves in the brain, releasing animal spirits to hollow nerve tubes that lead to appropriate muscles (see Fig. 1). This general outline of the reflex arc as the basic operation of the nervous system has remained immensely important, even if subsequent research rejected hydraulic metaphors.

The structure of the reflex arc is retained in most of the theoretical accounts of the nervous or psychological phenomena. For example, the understanding of the mechanism of behavior in terms of the reflex arc is both plausible and intuitive: the stimulus affects the receptor, which then causes the effector to respond. Needless to say, associative psychology also fits the reflex arc vision of the nervous system: the associations in the brain simply *are* reflex arcs. Similarly, it’s quite plausible to understand the reflex arc as an input-output processing system, to use the computational lingo. While the notion has been repeatedly criticized (e.g., Dewey 1896), it has remained immensely influential. It became the basis for the work on the nervous system for centuries to come, and its strongest proponent was probably Sir Charles Sherrington (1920). The sophisticated versions of the reflex arc assume that the previous state of the arc may modulate the response; in other words, the response to the same stimulus need not remain the same over time. There may be habituation or inhibition. However, in essence, the reflex arc shows the function of the nervous system: take in the incoming input, sensory information, and get the output, motor behavior. How could it be otherwise?

The skin-brain thesis is an attempt to sketch an alternative story of the evolution of the nervous system. It’s based on earlier proposals, in particular on the work of Carl Pantin (1952, 1956). Pantin argued that muscle coordination was the basic reason for the evolution of early nervous systems: animals such as actinians should not be thought to be ‘simple behavior machines’, whose functioning is based on simple direct reflexes. Rather, ‘the simple reflexes are only part of the whole behavior machines’ (Pantin 1952, p. 165). Note that Pantin’s idea is close in spirit to the constructivist biosemiotic theories: instead of assuming that semiotic systems are simply passive mirrors of nature, they are essentially entities that act in their environments, and they could need cognitive contact with the environment only later, to support their later evolution.

There are multiple reasons to be skeptical about the reflex arc as the basic building block of the nervous net. Keijzer presents clues that the reflex arc account is wrong. If the nervous system is built from such arcs, why should it be a *diffuse network* rather than just a bundle of pipelines? The functioning of the nervous system is much more complex than one time input-output responses (though, admittedly, a sophisticated reflex arc may admit some action modulation). But Keijzer points out more: first, the reflex arc may be a secondary optimization of the nervous system. Granted, there *are* reflexes but they are not the basic adaptation; they are more likely to be an exaptation. Moreover, there can be biological information processing without the nervous system: early organisms had no complete nervous system but only some nervous cells. Basic nervous systems do not lead to more complex behavior often displayed by organisms without a nervous system.

As far as the molecular level is concerned, many of the biomolecular characteristics of neurons are already present in non-neural precursor contexts. This includes ion channels, neurotransmitters, and synaptic protein families. So, Keijzer argues, understanding what nervous systems do is a question that requires an answer at the level of the whole animal. His understanding is clearly influenced by Pantin, as at the level of the whole animal, the main effector is the muscle tissue, and this tissue requires spatiotemporal coordination (note that other internal coordination models of early nervous systems, reviewed below, would also point out that at the level of the whole animal, multicellular organisms also require physiological control; cf. (Arendt et al. 2015)). This spatiotemporal coordination requires endogenous activity, if the animal is not supposed to be purely reactive.

What could then lead to such coordination? According to Keijzer, there is a half-station, a primitive conductive epithelium, enabled by chemical transmission between adjacent cells. This could have enabled muscle coordination in myoepithelia. The myoepithelium is an epithelium that also has contractile (myoid) properties, thought to be a precursor of nervous systems. What then? Specialized axodendritic connections can have subsequently evolved to broaden the existing possibilities for muscle coordination.

If we take all these clues into consideration, we may see the reason for supporting the basic claim:

early nerve nets evolved when some conducting cells—either within or connected to the myoepithelium—evolved elongated processes and synaptic connections in a way that modified and enhanced the patterning capabilities of a pre-existing myoepithelium (Keijzer et al. 2013, p. 80)

**Fig. 2:**
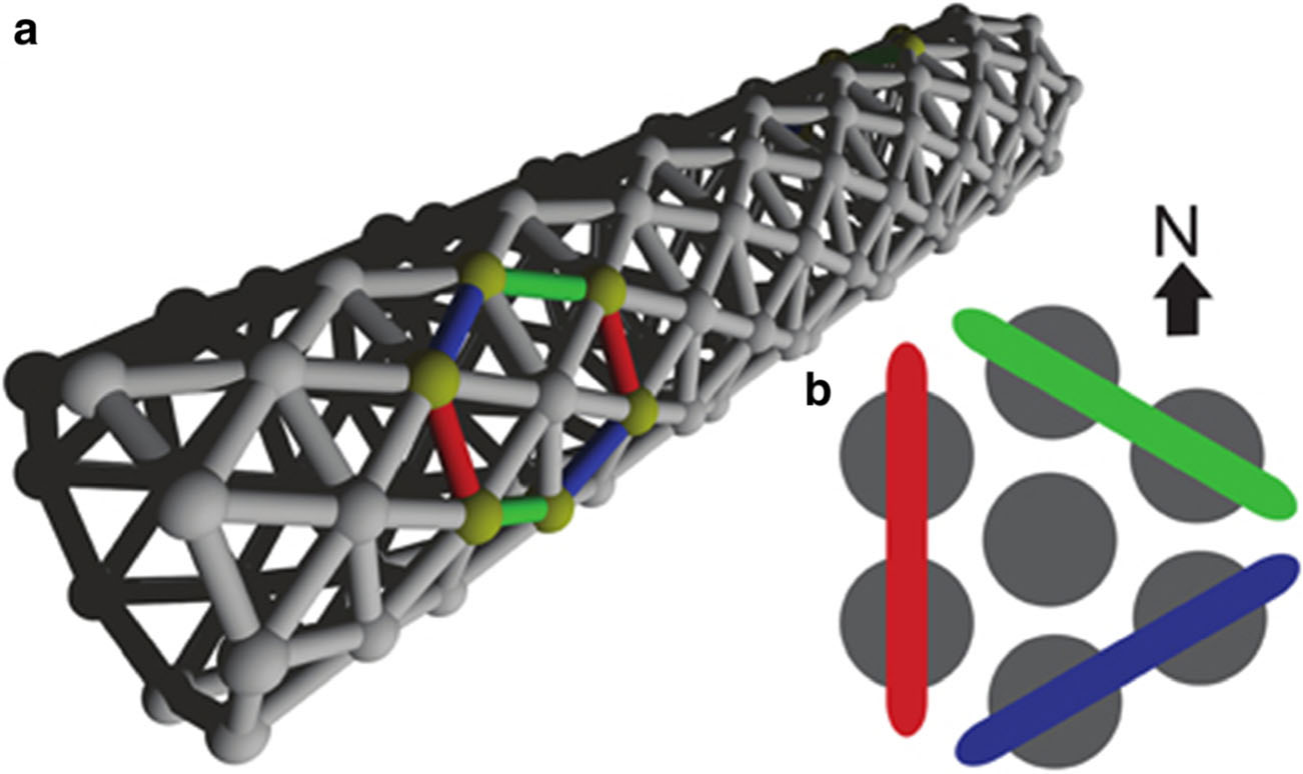
Model network built according to the skin-brain thesis. (**a**) The tube-shaped model animal. The epithelium consists of excitable cells arranged into a triangular lattice wrapped around a cylinder. (**b**) Color coding used to refer to the three differently oriented wave fronts on this lattice (Wiljes et al. 2015, figure available on CC BY license)

The skin-brain thesis is supported not only by generic considerations presented above but also by a computational model that suggests that “excitable epithelia using chemical signaling are a potential candidate as a nervous system precursor” (de Wiljes et al. 2015, p. 1). The model is a proof of concept, and it depicts a system ‘capable of self-generated activity patterns without explicit input from outside’ (de Wiljes et al. 2015, p. 3). An important factor in the model is spontaneous activity, and the purpose of modeling was to explore whether it could support self-organized whole body organization. The researchers conclude that a tube-shaped animal (see Fig. 2) with an appropriate epithelium could indeed display whole body coordination (the detailed assumptions need not concern us here). They conclude:

the generic properties of the modeled excitable epithelium enable a rudimentary form of coordinated patterning. This occurs without any sensory input, without any central pattern generators and without requiring any specific wiring or particular connections between the cells. Coordination can be cast as an ingrained self-organized feature of such a multicellular organization (de Wiljes et al. 2015, p. 9).

Note that this does not only satisfy criteria of the minimal cognition as spelled out by Barandiaran and Moreno; this model seems to include certain abilities to operate on information internally in a self-organized fashion, which will lead to classification of internal ‘objects’, so this will also be a minimal mind as defined by Sharov.

The basic message is that the nervous system evolved initially as a device to coordinate internal activity, enabling multicellular effectors. This is in line with other hypotheses about the evolution of the nervous system. The earliest proposal is due to Nicolaus Kleinenberg, a German biologist who obtained a doctorate degree under Ernst Haeckel (Kleinenberg 1872). He has identified a cell with two processes: sensory and muscular, and dubbed it ‘neuromuscular’. These neuromuscular processes had, according to Kleinenberg, all the components required for the reflex arc. The most influential however was the hypothesis offered by G. H. Parker, who has summarized his research in his now classic book *The Elementary Nervous System* (Parker 1919). Parker’s hypothesis is that the evolution proceeded in three successive phylogenetic steps: (1) *independent effectors*, represented today, according to Parker, by sponge myocytes or coelenterate nematocysts; (2) receptor cells, evolved from undifferentiated epithelium adjacent to the muscle cells; (3) protoneurons, evolved between receptor and effector cells to create the reflex arc. So while there is a considerable similarity at stage 2 of the development with the skin-brain thesis, Parker’s end result is supposed to be the dignified reflex arc. Interestingly, subsequent researchers, including Pantin, have not questioned step 1, even if Pantin stressed that the entire muscle sheet has evolved first, not just individual effector cells. As Passano summarizes Pantin’s position to Parker, “Pantin sees the nervous system evolving (without specifying in detail whence it came) in an integrated organism with a well-developed muscle sheet effector system” (Passano 1963, p. 307). Passano stresses that Pantin’s proposal is a significant improvement as it provides for endogenous activity within the primitive nervous system. Passano presents another hypothesis, close in spirit but more focused on dynamical and integrative properties of the organism:

We propose that individual protomyocytes first evolved into assemblages of independent contractile cells, permitting more extensive movements resulting from contractions of individual myocytes. Certain of these cells became endogenous activity centers, or *pacemakers*, by developing membrane areas capable of active depolarization. Such local pacemakers synchronized contractions of adjacent cells by passive depolarization spread affecting the contractile mechanism, perhaps utilizing intercellular bridges. Groups of muscle cells responding to pacemakers would permit the evolution of recurrent feeding movements. Differentiation of these would have thus proceeded together, with what was to become nerve specialized for contraction and what was to become the nerve specialized for activity initiation. Initially both would become specialized for passive conduction of depolarization. The specialization of the nerve cell for conduction rather than the repetitive initiation of activity is seen as a secondary development in the evolution of neurons (Passano 1963, pp. 307–308).

This idea was further developed by Mackie (1990), who suggested that the nervous system evolved from primordial myoepithelial-like cells, capable of contraction, transmission, and reception.

There are also alternative proposals. For example, Moroz suggests that there are multiple origins of nervous systems, and that “early secretory/peptidergic cells were evolutionary precursors of neurons and that the massive gene upregulation needed to repair an injury was co-opted to serve the needed neuronal integrative functions” (Moroz 2009, p. 187). A recent hypothesis defended by Arendt et al. (2015) is that the process of evolution of the nervous system began with distinct integration centers that evolved on opposite ends of an initial nerve net. The ‘apical nervous system’ controlled general body physiology, and the ‘blastoporal nervous system’ coordinated feeding movements and locomotion. Arendt et al. suggest that integration and fusion of these centers gave rise to the bilaterian nerve cord and brain.

To sum up, current theories may be divided into two large groups: input-output theories, following largely the reflex arc account; and internal coordination models, which “emphasize the role of nervous systems in coordinating multicellular activity, especially muscle-based motility” (Jékely et al. 2015). Jékely and colleagues stress that both models need to be taken into account in the broader view on the evolution of nervous systems, and they can be integrated in a single framework. The internal coordination models stress the control properties of the nervous system: they are supposed to have evolved for motor control (the skin-brain hypothesis), metabolic control and motor control (Arendt et al.), and injury repair (Moroz). Of course, sensory capacities are required at later stages, as well. However, this does not undercut the argument presented here: it’s quite obvious one needs to account for the role of sensory reception in the nervous system.

Most early nervous systems satisfy the requirements of minimal cognition, and they supply the organism with the information about its own motor states and physiological states. It does not seem to be a huge stretch to claim that this kind of information is the evolutionary precursor of what we call ‘knowledge’. In other words, a posteriori cognition is possible without sensory reception. This kind of research shows that it’s at least in principle possible to claim that such systems are minimally cognitive.

Note that this does not mean that current organisms have no receptor cells. This would be absurd. While some of the proponents of the hypothesis about the evolution of the nervous system pointed to contemporary marine organisms, the skin-brain thesis is not that all brains are like this. The claim could be still true even if all contemporary organisms had a different organization. So the claim is not about the current organization of the nervous system; it’s about its evolution. The evolutionary story only shows that the early stages need no sensory contact with reality to produce minimal cognition. At later stages, the nervous systems obviously attained the function of sensory reception. As Godfrey-Smith stresses:

Perhaps, then, the very first nervous systems primarily served to coordinate actions—first animating the body of an ancient cnidarian, then shaping the actions of Ediacarans. But if there was such an era, by the Cambrian it was over (Godfrey-Smith 2016, p. 39).

Like Feyerabend (1969), Fodor (1991), and Humphreys (2004), I want to deny (EE) by noting that *sensory* contact with reality is *unnecessary* for cognition, as it can be replaced with non-magical and non-mystical informational contact with reality. Feyerabend, Fodor and Humphreys all point to measuring instruments as producing knowledge; these need not be observed by anyone’s sensory apparatus to count as the origin of knowledge and justify claims about knowledge (at least according to the reliabilist account of knowledge, cf. (Goldman 1994)). I pointed to informational processes related to motor control in the myoepithelium. These are not environmental property detectors but they bear factual and instructional information (Floridi 2010) about action-related states. The reliability of such information would suffice, under the reliabilist account of knowledge, to justify knowledge claims regarding early nervous systems. In other words, early nervous systems are cognitive without sensory receptors because they both provide external justification for claims about cognition and produce cognitive states.

One could propose a refined version of (EE), however, by replacing “sense experience” with “property detection” (as suggested by Humphreys 2004), or, more generally, with “factual and instructional information”, but whether such formulation would count as empiricist is contentious because empiricists such as David Hume or Bas van Fraassen relied heavily on the primacy of sensation.

## Is Empiricism Empirically Falsified?

There are at least two possible objections to the argument presented above. First, it could be argued that I have picked an arbitrary notion of minimal cognition, and that cognition requires more than the early nervous system had. Second, it could be argued that just because there is a kind of negative feedback, one could say that there is sensory reception, if not experience, because there is a kind of epistemic contact with the environment. I will deal with these objections in turn.

### Minimal Cognition

Obviously, some proposals related to the notion of minimal cognition seem to presuppose a kind of reflex arc architecture (as Calvo Garzón along with van Duijn seem to do). If I adopted this kind of notion of minimal cognition, my argument would of course fail. But the notion defined by Barandiaran and Moreno seems to be quite well-motivated, and in line with other hypotheses about the evolution of the nervous system: the primary adaptive function of the said system is internal coordination and motor control. For this reason, this notion seems natural in this kind of argument. However, my opponent could easily reject it, by supplanting it with another notion of minimal cognition, or even argue that minimal cognition is not relevant.

I grant that my argument is not a silver bullet. However, in the broad field of naturalistic approaches to cognition, the rejection seems to be ill-motivated, especially for proponents of constructivist models of cognition and communication. Note that the proof of concept seems to satisfy also the requirements of the minimal mind as defined by Sharov.

### Sensory Reception

A much more difficult objection is based on the notion of sensory reception. One could argue that there is already sense reception in the skin-brain hypothesis (Fatima Cvrčková, personal communication); namely, the whole myoepithelium is both a sensory receptor and an effector (this would also apply to Parker’s original proposal, as it was based on “individual effectors”, and to Pantin’s refined hypothesis). A quick reply, namely that the epithelium is not classified as a sensory receptor in neurophysiology textbooks, won’t do. There are serious difficulties in defining what counts as a *sensory* receptor and what does not, so my reply has to cover more ground. Obviously, there may be other kinds of cellular receptors in the skin; the question is whether they are sensory or not. If not, their existence is also a source of trouble for (EE).

Basically, there are two ways of understanding sensory receptors; first, as related to a specific functional pathway over which the information is transmitted, second, as performing a function of registering the sequence or arrangement of stimulation, or information. I will discuss both in turn.

The traditional notion of the receptor is entrenched in the “law of specific nerve energies”, as defended by Johannes Müller. The law states that the nature of sensation is defined by the specific energy of the stimulus. In its original wording, the law is outdated, as it supposed that there are different specific energies that individuate the senses; hence, according to Müller, receptors capable of sensing electricity are a priori impossible (Müller 1842, p. 1087). So the only way to create a new sense, according to Müller, would be for the external cause to excite a new kind of energy, different from the five known senses. In its modern formulations, the law is supposed to say that a sensory modality is not defined by the origin of the excitation but by the pathway or the target brain area. It’s reinforced by observations of Edgar Adrian that neural pathways conduct electricity, so the basic bearer of all sensations is the same – signals have the same form but are received by different parts of the brain (Adrian 1947).

As is quite clear, with this understanding of sensory receptors, one could easily deny that the myoepipithelium is a sensory receptor. There is no specialized location for signal reception, so even if these receptors transmit signals, the signals are not sensory, however. This kind of rebuttal may sound hollow, I’m afraid, for the same reason as Müller’s rejection of electroreceptors in fish sounds ridiculous. All the worse, brain plasticity seems to imply that sensory substitution is possible: blind people are able to use their touch sense to detect visual stimuli (to a limited degree). This flies in the face against the specific energy doctrine (Kiverstein et al. 2014). Moreover, non-blind people learning to read Braille *with their hands* use their “visual” cortex to read, which means, according to some neuroscientists, that there is no sensory quality specialization that would allow to distinguish the senses (Siuda-Krzywicka et al. 2016).

These facts seem to suggest that another way of understanding the sensory reception is more plausible, a functional one, defended by J. J. Gibson in his now classic work on the perceptual systems (Gibson 1966). As he notes: “there is not a specific nerve for each sense despite a popular idea to the contrary” (Gibson 1966, p. 41). Note that Gibson distinguishes sensory organs and sensory receptors. The organs are active systems for perception, mobile, exploratory, orienting, while receptors are “immobile” parts of the input system: “What they register is not the energy of the stimulation, but the sequence or arrangement of stimulation, that is, information” (Gibson 1966, p. 41). By referring to Gibson, one could perhaps define sensory reception functionally, as the capacity for receiving information. He also notes that Sherrington stressed that specialization of the receptor involves that it “has a low threshold for a particular kind of energy and a high threshold of all other kinds” (Gibson 1966, p. 43). But this would be true of many, if not all, causally interacting systems. This broad definition would cover any kind of negative feedback, and hence, would be detrimental to the argument presented herein.

But the Gibsonian account is much more complex. Sensory receptors are just parts of the perceptual systems, and are defined by their role in the system: “organs of sensitivity, like other organs of the body, exist in a hierarchy of organization. Lower organs are subordinated to higher. Smaller structures serve larger structures, and they overlap. The eyeball is ‘all of a piece,’ but it is an unusual sense organ.” (Gibson 1966, p. 42) The function of the perceptual systems is to pick up the environmental information, and just because Gibson includes proprioception, the environment may as well be internal. Hence, the Gibsonian account of sensory reception seems to be the strongest line of defense for the empiricist. After all, he also expounds a version of it: “All knowledge rests on sensitivity” (Gibson 1966, p. 26).

But do models like the one described by the skin-brain thesis really appeal to sensitivity? To answer this question, I will first distinguish information-based causation, and then expose one important feature of sensory reception, namely that it should be able to support perceptual constancies and detection of distal objects.

The interaction of biological systems with their environment need not be only informational. Solar energy does not only incite rods and cones in human eyes but also influences the body heat. In other words, the organism may stand in a non-informational interaction with the environment without any sensory reception, or more generally, without any information detection. This is not to say that causation is not the transfer of information; on the contrary, it is (Collier 1999). But only some transactions of the organism with the environment have the function of information transfer.

There are different proposals as to how to differentiate information-based causation from other kinds of causation. One could, for example, require that the information in question be semantic, or, to be about something. This requirement may be spelled out in various terms; for example, one could require that the causal factors be true-evaluable meaningful signs. Or one could use a fairly minimal notion of information as *structural*, or *logon* information (MacKay 1969). For a medium to carry structural information, it is necessary that it has at least two differentiable states. A stream of structural information is a sequence of states of the information medium. In our biological case, if anything is to serve the role of the information medium, its states have to be differentiated by some detector, or the difference between the states has to be causally efficient. There may be physical differences in the biological medium that have no bearing for the informational nature of the medium. For example, DNA may be considered an informational medium in the structural sense but the biological machinery, as far as we know, is not sensitive to the fact whether the DNA strand finds itself in the very center of Warsaw or 100 km away from it, even if it’s a physical fact that it has a certain spatiotemporal location.

If we assume that receptors produce sequences of information states, then the state changes caused by them in the organism should be explainable in an informational-structural fashion. This may be spelled out in terms of substrate neutrality (Dennett 1995; Piccinini 2015): the exact physical nature of the bearer is causally irrelevant for the information transaction to happen. This is *not* to say that no physical properties matter, it’s just that they are not a part of a proper causal explanation. So while the DNA strand has a certain geographical location, this geographical location will not figure in the proper explanation of transcription etc. But, in principle, as far as the information-structural causation is concerned, the DNA parts could be replaced by some other physical substrate *if* it preserves all the relevant causal topology. Similarly, it does not matter whether you store your data on a CD-ROM, a USB disk, or a punch card, as long as you have (different kinds of) a physical machinery that responds to different states in the information media in the equivalent fashion. Of course, you cannot replace any part of the USB disk with any part of a CD-ROM, and expect its work to continue. But you could substitute the whole medium if you have an appropriate responding machinery (or a *consumer* in Millikan’s (1984) terminology) in place, with the same causal output profile.

All proper causal explanations are contrastive (Hitchcock 1996; Northcott 2008). For example, you cannot properly explain why my body temperature has lowered if you say that I took one pill of aspirin. The proper explanation should specify that this amount of aspirin – and *not* the other – caused my temperature to become lower *this* way, and *not* another. In informational-structural causation the contrast classes cite only physical features of the medium that distinguish the information states of the medium as recognized by the receiving machinery.

Now we can go back to the notion of the sense receptor; it needs to pick up information, so it should not only be sensitive to any causal factors but just to physical informational states of the information media. Solar heat influences my body temperature directly, and the proper explanation of this process will not refer to contrast classes in my skin, body fat and muscles etc., *as bearing any structural information*. In contrast, the rods and cones in my retina change their states systematically with the environment information as available in light. The skin-brain model does not have any sense receptors in this sense; while the tube-shaped body will react directly with the external environment, it will not react in the way that could be properly described in terms of the informational causation. There is no sufficient *systematic* relationship with the environment for this. Of course, the motor effectors will respond in a way that should be described informationally but *not* to the states that are caused informationally by the external environment.

My opponent could now argue that the neurons in the skin-brain model act similar to proprioception: the whole body could be considered as a huge receptor (and indeed, Gibson (1966, p. 43) says that the ameba “is all one receptor”). But the modelers stress that the whole body coordination occurs without “sensory input”. Something’s got to give. And it’s arguable that the Gibsonian claim is just a metaphor: ameba, were it “all one receptor”, would have to be literally just a passive organism. Amebas, in contrast to many other unicellulars, have no dedicated sensory receptors, this is true, and they are discriminative of sensory stimuli. But they also display coordinated behavior, so they have the perceptual system that makes use of the stimuli, and this is not passive in the relevant sense. In contrast, the skin-brain model has no discriminative abilities with regards to its external environment, and the internal coordination does not seem to imply any *sensory* detection worthy of its name. This is because there is no ecological meaning of the internal signals, and the proximal signals do not seem to carry any *distal* information even about the Innenwelt of the skin-brain animal. In other words, there are no properties, as far as we can observe, that could support perceptual constancy about any objects of the sensory receptors (which is required for proper information registration in animals; cf. (Burge 2010)).

To sum up, the first objection seems to be fairly weak. The second one opens up an interesting line of inquiry; still, the argument seems undefeated. Without trivializing the notion of the sensory receptor to a causal factor responsible for physiological, behavioral, or developmental changes, one cannot really make this move. First, the states of the skin-brain model are not *informationally receptive* in the appropriate ways to serve as receptors of external information; second, these states are not *sensory*, as they could not have any perceptual meaning.

## Summary

In this paper, I sketched the discussion over the evolutionary function of nervous systems, especially their origin. My purpose was to show that this discussion has a bearing on philosophical and theoretical discussion of important claims. The empiricist account of meaning (EM) was not my focus because most if not all formulations of (EM), in particular in terms of empirical verification, turned out to be hopelessly problematic (Hempel 1950, 1951; Zabłudowski 1966a, 1966b). The argument presented above, however, is not aimed to undermine (EM), as the mere possibility that there might be non-sensory synthetic a posteriori knowledge does not imply that the meaning of all expressions of human natural languages cannot be reduced to the meaning of observational statements.

The above discussion has consequences for epistemological empiricism (EE), however. If some internal-coordination models of the evolution of the nervous systems are valid, then it’s plausible to think that (EE) is falsified or unmotivated. Let’s look at both possibilities in turn.

First, if you think that (EE) is a conceptual claim, then it should be impossible to describe a cognitive system without sensory experience. But it seems that there could be minimally cognitive systems devoid not only of sensory experience, but also of sensory reception or input altogether. This is what internal-coordination models of early nervous systems show. So either these models are contradictory (but where exactly?), or they smuggle sensory reception somewhere. I pointed out that the organism may react with the environment directly, and be influenced by it passively, without having sensory receptors, and still gather some internal information patterns useful for its own body coordination. In a minimal sense, this kind of system is cognitive, and has a posteriori knowledge about itself.

But there are problems with understanding (EE) as truth-evaluable because (EE) is itself not justified with the senses; at best, it could be seen as a fallible inductive inference, as we haven’t seen yet a counterexample (but, as I point out, early nervous systems may be such counterexamples). So one could state (EE) as a motivated stipulation. The stipulation turns out to be ill-motivated, as there are reasons to think that sensory reception, not to mention sensory experience, is not essential to a posteriori knowledge. Obviously, one could respond that (EE) glosses over early and minimally cognitive systems, and full-blown cognition requires sensitivity after all. This is a sound response but a sounder one, for an empiricist, is to reject the reliance on the senses, as Feyerabend and Humphreys suggested in their arguments. In other words, the intention of this paper is to overthrow the epistemic primacy of sensation. Not only measuring instruments can both produce and be warrants of knowledge in appropriate mechanisms; also early nervous systems can produce and warrant reliable knowledge.

More generally, this also shows that constructivist accounts of semiotics and cognition, as opposed to some forms of empiricism (of course, there are constructive empiricists who also expose idealism or antirealism, to mention just Berkeley), may seem plausible. Early nervous systems had first to make sense of themselves, and only later evolved sensory systems that would inform their own construction. In other words, while knowledge without sensation is possible, it does not mean that sensory receptors are useless. On the contrary, both sensory receptors and other specialized property detectors, such as measuring instruments, are crucial in attaining knowledge. But they are not strictly necessary.
